# Influence of larval density and dietary nutrient concentration on performance, body protein, and fat contents of black soldier fly larvae (*Hermetia illucens*)

**DOI:** 10.1111/eea.12716

**Published:** 2018-09-03

**Authors:** Karol B. Barragan‐Fonseca, Marcel Dicke, Joop J.A. van Loon

**Affiliations:** ^1^ Laboratory of Entomology Wageningen University PO Box 16 6700 AA Wageningen The Netherlands; ^2^ Departamento de Producción Animal Facultad de Medicina Veterinaria y de Zootecnia Universidad Nacional de Colombia Bogotá Colombia

**Keywords:** detritivory, diet quality, growth, nutritional value, ration, survival, Diptera, Stratiomyidae

## Abstract

Performance and body composition of insect larvae depend on quality and quantity of their diet, and on biotic factors such as larval density. We investigated the effect of dietary nutrient concentration and larval rearing density on survival, development, growth, and protein and fat contents of larvae of the black soldier fly (BSF), *Hermetia illucens* L. (Diptera: Stratiomyidae). Neonate larvae were fed with a low (NC1), intermediate (NC2), or high nutrient concentration (NC3), and with four rearing densities (50, 100, 200, or 400 larvae per container). Two feeding regimes (FR) were tested: in FR1, the amount of diet added during the experiment was based on the visually estimated larval mass present, whereas in FR2, a fixed feeding ration of 0.6 g of food per larva was applied at the start. FR1 resulted in food limitation, resulting in significantly lower body crude protein content on diet NC1 than on NC2 at larval densities 100 and 200. Larval crude fat content was higher on diets with higher nutrient concentration and at lower larval densities. For FR2, development time was shorter on diets with higher nutrient concentration and at lower larval densities. Individual larval weight and total larval yield increased with higher nutrient concentration at all four larval densities. At lower nutrient concentration, higher larval density resulted in higher individual larval weight and total larval yield, revealing an interaction between larval density and dietary quality. Larval crude protein content was higher at lower densities and lower nutrient concentration. Larval crude fat was higher at higher larval densities and nutrient concentrations. This study indicates that larval protein content is regulated within narrow limits, whereas larval crude fat content is strongly affected by nutrient concentration and by larval density.

## Introduction

The size to which an individual insect grows is affected by both genetic and environmental factors that operate through complex molecular and physiological mechanisms (Nijhout, 2003). Growth rate varies substantially in response to various stimuli, including resource availability, competition, predator presence, time of season, humidity, and temperature (Scriber & Slansky, 1981; Harnden & Tomberlin, 2016). Moreover, food quality affects many life‐history traits such as larval and adult performance (Moreau et al., 2006).

High food quality enhances the rate of development and increases survival in some insect species (de Haas et al., 2006). For example, Nguyen et al. (2013) and Oonincx et al. (2015b) observed that the detritivorous larvae of the black soldier fly (BSF), *Hermetia illucens* L. (Diptera: Stratiomyidae), when fed diets of vegetable by‐products high in protein, had a shorter development time (21 days) than larvae fed low‐protein diets (37 days). Conversely, Simon et al. (2011) suggested that diets with a higher proportion of protein increase development time and survival rate of some predatory fly species.

Nguyen et al. (2013) established an increase in growth rate and decrease in developmental duration of BSF larvae on both high‐protein and high‐fat diets. However, indications have been found that high levels of fat – 20–36% crude fat based on dry matter (DM) – may be detrimental for both larval and adult survival, lifespan, and reproductive output (Ujvari et al., 2009; Nguyen et al., 2013). It seems that availability of balanced amounts of calories, fat, and protein may be more important for fast development and higher larval weight than only a high‐protein content (Nguyen et al., 2013).

Although BSF larvae on average contain both a high‐protein and a high‐fat level compared to other edible insect species (Zheng et al., 2012a; Barragan‐Fonseca et al., 2017), body composition of the larvae depends on the quality and quantity of ingested food (Nguyen et al., 2015; Oonincx et al., 2015a,b). Consequently, larvae fed on different substrates had varying body protein (ranging from 37.0 to 62.7% DM) and fat content (6.6–39.2% DM) (Barragan‐Fonseca et al., 2017).

Also physical factors may influence insect performance. For example, if the layer of food substrate consisting of meat meal, swine meat, fish, or liver is too thick, larval food intake is reduced resulting in lower survival and longer developmental time (Nguyen et al., 2013). Lardé (1989) fed BSF larvae on coffee‐pulp substrates with different composition, and observed that BSF larvae grow better on the most homogeneous, dense, and dry substrate (780 g kg^−1^ wet matter solids). Moisture levels of 60–70% in manure and chicken feed have been found adequate for BSF larvae (Fatchurochim et al., 1989; Tomberlin et al., 2002; Myers et al., 2008; Holmes, 2010). However, due to the variable composition of organic waste, food moisture is difficult to control and needs to be evaluated not only under laboratory but also under field conditions where evaporation rate fluctuates.

Biotic factors may also affect BSF performance. For example, larval density can be a major factor affecting the rate of development (Tomberlin et al., 2002; Diener et al., 2009). Parra Paz et al. (2015) demonstrated that larval density has a significant influence on bioconversion of residual organic matter into body mass by BSF. BSF larvae tend to aggregate and overcrowding slows larval development due to competition for feed (Rivers & Dahlem, 2013). Moreover, high larval densities may result in decreased substrate quality by accumulation of larval waste products (Green & Popa, 2012) and may generate direct energetic costs if larvae spend extra energy interacting with each other (Jannat & Roitberg, 2013).

Compensatory mechanisms are activated in response to crowding and nutritional deficiencies. Insects have a tendency to prolong the larval period (Miller, 1964), or to increase either the rate of ingestion or the total amount of food ingested during larval development (Green et al., 2003). Sullivan & Sokal (1963) proposed two basic types of responses to crowding: (1) a reduction in the number of individuals able to complete their life cycles, with the emerging adults maintaining normal body size, and (2) sustaining survival accompanied by reduction in body weight, as has been reported for the dipterans *Phormia regina* (Meigen) (Calliphoridae) (Green et al., 2002), *Drosophila melanogaster* Meigen, *Drosophila simulans* Sturtevant (Miller, 1964), and *Aedes albopictus* Skuse (Yoshioka et al., 2012). According to Sullivan & Sokal (1963), for the second type of response substantial losses in numbers will occur only at densities above which adult weight drops below a critical limit.

Lower larval densities are not always better to maximise growth rate. In some insect species, larval aggregations provide adaptive benefits to individuals due to heat generation, which might enhance food assimilation (Green et al., 2002) and provide protection from low environmental temperature and possibly predators (Rivers & Dahlem, 2013). BSF larval weight gain is also affected because of their potential dependence on bacteria as food (Liu et al., 2008). Higher larval densities are associated with higher bacterial densities which might allow larvae to have better access to bacterially recycled nutrients, thereby resulting in more effective nutrient absorption. Therefore, optimising density may benefit the productivity of insect rearing.

Detailed knowledge of the conditions required for optimal growth, development, and nutrient allocation of BSF is necessary for implementation of large‐scale production systems (Coelho et al., 2013). The effect of nutrient density of the ingested food on development, growth, and body composition of BSF in interaction with larval density has not been investigated systematically before. The aim of the present study was to investigate the effect of dietary nutrient concentration (NC), larval rearing density, and the possible interaction between these two factors on growth characteristics and nutritional composition of BSF larvae.

## Materials and methods

### Experimental insects

*Hermetia illucens* larvae were obtained from a colony maintained under constant conditions in a climate room (27 ± 1 °C, 70% r.h., and L12:D12 photoperiod) at the Laboratory of Entomology, Wageningen University, The Netherlands.

### Experimental design

This study was based on a 3 × 4 factorial design with three levels of dietary nutrients (NC1‐3) obtained by diluting commercial chicken feed (Opfokmeel Farmfood; AgruniekRijnvallei Voer, Wageningen, The Netherlands) with cellulose (Alphacel non‐nutritive bulk; MP Biomedicals, Illkirch, France) (Table 1), and four rearing densities (D50‐400) obtained by placing 50, 100, 200, and 400 larvae (<24 h since hatching) per plastic container (15.5 × 10.5 × 6 cm) resulting in 0.31, 0.62, 1.23, and 2.47 larvae cm^−2^, respectively. The expression of density per unit of surface is a proxy for actual relevant density, which should be expressed per unit of volume. The densities were selected according to the minimal and maximal densities calculated from information presented in studies on BSF performed by Sheppard et al. (2002). The plastic containers were covered with transparent plastic lids with 90 holes (0.05 cm diameter each) for ventilation.

**Table 1:** Composition of experimental low (NC1)‐, intermediate (NC2)‐, and high‐nutrient concentration (NC3) diets tested in feeding regimes 1 and 2

	NC1	NC2	NC3
Ingredient (%)			
Chicken feed	23	43	85
Cellulose	77	57	15
Nutrient (%)			
Protein	3.5	7	14
Fat	0.7	1.4	1.8
Non‐cellulose carbohydrate	12	23	46

Two experiments tested two feeding regimes (FR1 and FR2). In FR1, the amount of diet added per occasion (3× per week) was based on the visually estimated larval mass present in the same way as in a previous study (Oonincx et al., 2015b). This regime aimed to adjust the amount of diet added at a regular basis in such a manner that starvation was prevented while avoiding overfeeding. Overfeeding putatively leads to waste of diet due to profuse microbial growth. On each occasion an amount of diet that was visually estimated to meet these requirements was weighed and added, all replicates of the same treatment receiving the same amount of food as the replicate that required the largest amount. For each gram of food provided to the larvae, 1.6, 2.6, and 3 ml of tap water was added for NC3, NC2, and NC1 diets, respectively, to account for the greater water absorption by higher amounts of cellulose, to obtain ca. 70% of moisture.

In FR2, there was a fixed food ration of 0.6 g of food (dry matter basis) per larva (density‐independent food availability), which was provided at the beginning of the experiment. A preliminary experiment had shown that larvae fed 1× rather than 3× per week reached a higher biomass. For each diet, six replicates were performed. One replicate was one container with either 50, 100, 200, or 400 larvae. Both feeding experiments were conducted in a climate room (4.5 m^2^) at 27 ± 1 °C, 55 ± 5% r.h., and L12:D12 photoperiod. In order to eliminate possible effects of position in the chamber, all containers were randomly relocated 3× per week.

### Immature life‐history traits

All larvae in a container were harvested when the first prepupa was observed, distinguished by the characteristic black cuticle, contrasting with the white larvae (May, 1961). All animals from each container were harvested with forceps, and counted. Larvae were washed under running water to remove feed and faecal residues, their integument was dried with paper tissue. To obtain dry mass, the samples were oven‐dried at 70 °C until constant weight. BSF larval yield (g DM) was determined using an Adventurer Pro AV313 precision balance (precision ± 0.001 g; Ohaus, Parsippany, NJ, USA). To determine survival rate, the number of live BSF larvae at the end of the experiment was divided by the initial number of larvae per replicate. Development time was considered to be the number of days between the start of the experiment and the observation of the first prepupa.

### Proximate chemical analysis of larvae

BSF samples were stored in a freezer (−25 °C) after dry mass had been assessed until all replicates were harvested. Both the larvae and the diets were analysed for dry matter, crude protein, and crude fat at the Animal Nutrition Laboratory of Wageningen University. The samples were oven‐dried at 70 °C until constant weight and homogenised by grinding the sample in a ZM 200 ultra‐centrifugal mill (Retsch, Haan, Germany). Nitrogen content was determined using the Kjeldahl method (ISO 5983‐1, 2005) and converted to crude protein content by multiplication with factor 6.25. Crude fat was analysed according to the Berntop method (ISO 6492, 1999). All protein and fat contents reported are on DM basis.

### Statistical analysis

Data were analysed with Generalised Linear Models to test the effect of nutrient concentration and rearing density as the two factors. To compare the effect of nutrient concentration within a larval density, one‐way ANOVA was performed (α = 0.05), followed by post hoc Tukey tests. Kruskal–Wallis tests were performed when assumptions of normality and/or homoscedasticity were not met. IBM SPSS Statistics v.21.0 was used for all analyses (IBM, Armonk, NY, USA).

## Results

### Feeding regime 1

#### Performance

**Figure 1:**
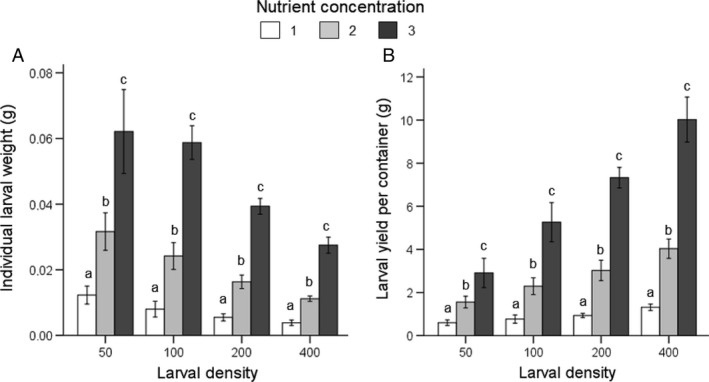
Black soldier fly mean (± SD) (A) individual larval weight (g dry matter) and (B) larval yield per container (g dry matter), at various larval densities and nutrient concentrations (feeding regime 1; see Table 1 for diet composition). Means within a density level capped with different letters are significantly different (D100 and D200: ANOVA followed by Tukey post hoc test; D50 and D400: Kruskal–Wallis test, all P<0.05).

Survival rate was affected by rearing density and density*nutrient interaction (GLM: P<0.001) but only marginally by nutrient concentration (P = 0.064) (Tables 2 and S1). Development time, individual larval weight, and larval yield (DM basis) were affected by nutrient concentration, rearing density, and their interaction (GLM: P<0.001; Table S1). Overall, development time increased with lower protein content. At densities D200 and D400 fed on the low‐nutrient diet (NC1) no prepupae were observed until 45 days after the start of the experiment (Table 2). Individual larval weight was higher at higher nutrient levels at each larval density (Figure 1A). Total larval yield per container was higher at higher nutrient levels (Figure 1B).

**Table 2:** Mean (± SD; n = 6) survival rate and development time of *Hermetia illucens* larvae under two feeding regimes (FR1 and 2) on three diets differing in nutrient concentration (NC), kept at four larval densities per container

Diet		FR1			FR2	
Density	NC	Food added (g/larva)	Survival rate (%)	Development time (days)	Survival rate[Link] (%)	Development time[Link] (days)
50	1	0.24	97.7 ± 2.5e[Link]	22.5 ± 1.5e[Link]	87.3 ± 4.1e	15.3 ± 0.5e
	2	0.41	98.1 ± 2.7e	16.7 ± 0.9f	93.0 ± 3.3ef	13.7 ± 0.8f
	3	0.51	93.6 ± 4.1f	15.9 ± 0.3f	95.0 ± 5.0f	13.3 ± 0.5f
100	1	0.14	95.5 ± 2.1g[Link]	26.8 ± 3.1g[Link]	92.8 ± 3.4g	15.7 ± 0.5g
	2	0.25	94.7 ± 2.7g	16.7 ± 1.0h	90.2 ± 4.8g	13.8 ± 0.4h
	3	0.36	89.5 ± 6.4h	15.8 ± 0.4h	92.0 ± 4.9g	13.5 ± 0.5h
200	1	0.09	87.1 ± 6.9i[Link]	–a	90.0 ± 4.7i	16.7 ± 0.5i
	2	0.17	92.0 ± 4.9i	21.0 ± 0.6i[Link]	90.5 ± 2.9i	15.7 ± 1.4ij
	3	0.2	93.9 ± 3.5i	15.8 ± 0.4j	89.2 ± 2.1i	13.8 ± 0.4j
400	1	0.06	88.2 ± 3.5k[Link]	–a	93.2 ± 3.7k	17.8 ± 1.3k
	2	0.1	90.5 ± 4.9k	21.5 ± 0.8k[Link]	88.7 ± 2.1k	17.0 ± 0.6kl
	3	0.13	91.7 ± 1.0k	19.2 ± 1.5k	92.8 ± 2.8k	15.3 ± 1.6l

Means within a column and within a larval density followed by different letters are significantly different (P<0.05). Comparison of means performed by ^1^Kruskal–Wallis test, ^2^ANOVA followed by Tukey post hoc tests, or ^3^Student's t‐test.

a No prepupae were observed until 45 days after the start of the experiment.

#### Larval body composition

**Figure 2:**
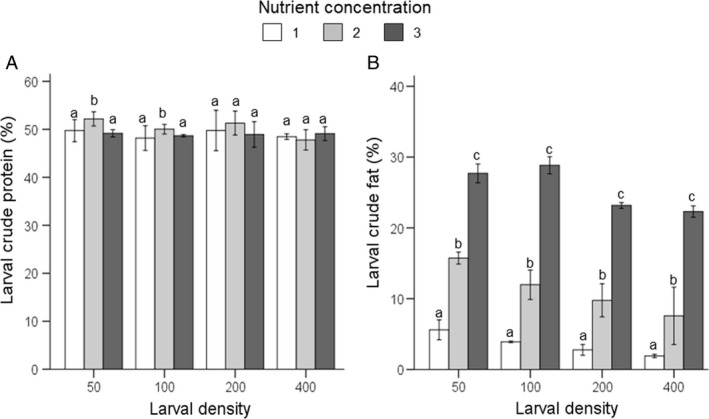
Black soldier fly mean (± SD) larval crude (A) protein (% dry matter) and (B) fat contents (% dry matter), at various larval densities and nutrient concentrations (feeding regime 1; see Table 1 for diet composition). Means within a density level capped with different letters are significantly different (crude protein, D50‐400 and crude fat, D50 and D100: ANOVA followed by Tukey post hoc test; crude fat, D200 and D400: Kruskal–Wallis test, all P<0.05).

Larval crude protein content was affected by nutrient content of the diet (F_2,24_ = 6.3, P˂0.001), rearing density (F_3,24_ = 6.3, P˂0.001), and their interaction (F_6,24_ = 2.7, P = 0.039). At D50 and D100, crude protein content was slightly higher for larvae fed NC2‐diet (D50, ANOVA; D100, Kruskal–Wallis test: both P˂0.05; Figure 2A, Table S1). Also crude fat content was affected by dietary nutrient content (F_2,24_ = 71.9, P˂0.001), rearing density (F_3,24_ = 30.2, P˂0.001), and their interaction (F_6,24_ = 2.9, P = 0.026; Table S1). Crude fat was clearly higher in BSF larvae fed NC3 compared to the lower two nutrient concentrations and higher for larvae fed NC2 compared to NC1 (Figure 2B).

### Feeding regime 2

#### Performance

**Figure 3:**
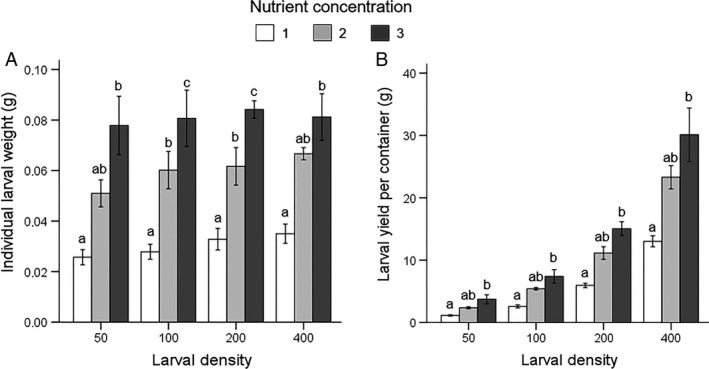
Black soldier fly mean (± SD) (A) individual larval weight (g dry matter) and (B) larval yield per container (g dry matter), at various larval densities and nutrient concentrations (feeding regime 2; see Table 1 for diet composition). Means within a density level capped with different letters are significantly different (larval weight, D100 and D200: ANOVA followed by Tukey post hoc test; larval weight, D50 and D400, and larval yield, D50‐400: Kruskal–Wallis test, all P<0.05).

Larval survival rate was not affected by either rearing density (F_3,60_ = 0.84, P = 0.48), nor by nutrient concentration (F_2,60_ = 1.2, P = 0.31) or their interaction (F_6,60_ = 2.62, P = 0.25) (Tables 2 and S1). Overall, larval performance was positively affected by both higher nutrient concentration and larval density. Development time was affected by nutrient content and rearing density (both GLM: P<0.0001), but not by their interaction (P = 0.12; Table S1). Lower densities and higher nutrient content accelerated larval development (Table 2). Individual larval weight and larval yield per container were affected by nutrient content, rearing density, and their interaction (all GLM: P<0.0001; Figure 3A). Higher nutrient concentration resulted in significantly higher individual larval weight for D100 and D200 densities. Larval yield per container increased at higher nutrient levels and densities (Figure 3B).

#### Larval body composition

**Figure 4:**
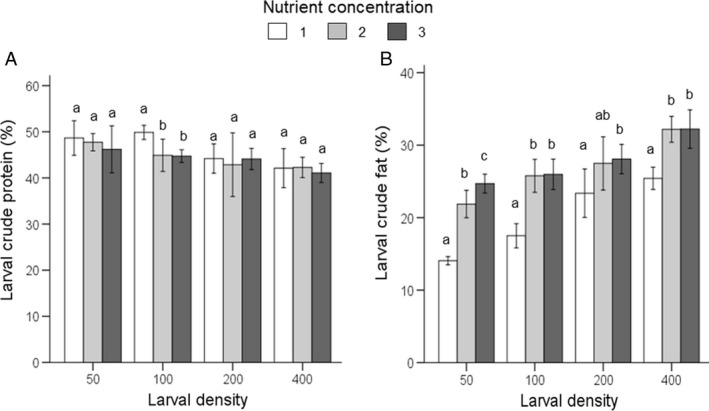
Black soldier fly mean (± SD) larval crude (A) protein (% dry matter) and (B) fat contents (% dry matter), at various larval densities and nutrient concentrations (feeding regime 2; see Table 1 for diet composition). Means within a density level capped with different letters are significantly different (ANOVA followed by Tukey post hoc test: P<0.05).

Crude protein content was affected by nutrient concentration (F_2,54_ = 8.5, P˂0.001), rearing density (F_3,54_ = 32.1, P˂0.001), and their interaction (F_6,54_ = 3.4, P = 0.006; Table S1). Overall, crude protein content in BSF larvae was lower at higher larval densities. Crude protein content was similar among NC‐levels per larval density; only at D100, crude protein content was higher for larvae fed on NC1‐diet (ANOVA: P˂0.05; Figure 4A). Crude fat was affected by nutrient content (F_2,54_ = 67.1, P˂0.001), and rearing density (F_3,54_ = 48.9, P˂0.001), but not by their interaction (F_6,54_ = 1.9, P = 0.096; Table S1). Larval crude fat was lower at NC1‐diet at all four densities (ANOVA, P˂0.05), and similar at NC2 and NC3 at each density except for D50 at which larval crude fat was significantly higher at NC3 (ANOVA, P˂0.05) (Table S1, Figure 4B). There was a significant relationship between larval weight and larval crude fat content (Pearson correlation coefficient r = 0.66, P˂0.005).

## Discussion

Nutritional strategies used by an individual or group of insects are shaped by their physiology, behaviour, and performance (Lihoreau et al., 2015). The processes of seeking, obtaining, utilising, and allocating nutrients are influenced by several biotic and abiotic factors, such as insect density and nutrient concentrations. Studying the effect of these factors on nutrition and development of insects is particularly important for mass‐rearing insects for feed and food in order to optimise productive performance and nutritional quality.

The results for BSF larval performance differed between the two feeding regimes. In FR1, it turned out to be problematic to visually estimate how much of the diet had been consumed, in particular for the low‐ and medium‐nutrient concentrations. This was caused by the high content of cellulose, added as non‐nutritive bulk to dilute nutrients. Only after collecting all results we could deduce that this had caused food limitation. Insects can respond to reduced nutrient levels in their diets by increasing either the rate of ingestion or extending the duration of ingestion (Slansky & Scriber, 1985; Slansky & Wheeler, 1989). During food shortage or other unfavourable conditions, BSF larvae reduce food intake or cease to feed (Diener et al., 2011a). As we did not measure food intake directly, we cannot distinguish which of the two responses occurred. Therefore in FR2, we supplied larvae diet ad libitum to properly assess the separate effects of nutrient concentration and larval density.

BSF larvae are extremely resilient due to their capability of dealing with unfavourable conditions (Diener et al., 2011b). This is confirmed by the high survival rates we observed, not only in FR2 but also in FR1, despite the strong food limitation in the latter regime at the lowest nutrient concentration and highest larval densities. The data from this study indicate that BSF larvae, similar to *D. melanogaster* and *D. simulans* larvae, are capable of physiological adjustments which allow them to survive in nearly equal proportions at low and high larval densities (Miller, 1964).

Despite the high survival rate, under extreme food limitation BSF larvae had extended development time (ranging from 16 to 27 days) or could not complete larval development at all. In FR1, the larvae with the strongest food limitation at higher densities (D200 and D400 at NC1) did not reach the prepupal stage until 45 days, after which observations were stopped. The increase in developmental period upon crowding is apparently related to the inability of the larvae to complete larval development under nutrient limitation.

In FR2, development time was affected by NC and larval density – increased with lower protein content and higher density – a pattern similar to that observed by Green et al. (2003) in larvae of *P. regina*. Larvae ingested food for a longer period of time, increasing their developmental time to obtain sufficient nutrition for pupation. The shortest development time (13 days) was achieved at the highest NC and lowest density, very similar to the time reported for BSF larvae fed with chicken feed (Spranghers et al., 2016).

Although for FR2 feed availability was density‐independent, high larval densities still resulted in prolonged development time. Probably, larvae have to spend more time to locate food or overcrowding may result in toxic concentrations of by‐products (Green & Popa, 2012; Yoshioka et al., 2012). Under crowded conditions, BSF has a tendency to prolong the larval period in a similar way as has been suggested to occur for *Drosophila* spp. (Miller, 1964), *Aedes aegypti* L. (Couret et al., 2014), and *Anopheles gambiae* Giles (Jannat & Roitberg, 2013). A compound that retards growth is produced by overcrowded larvae of these mosquito species that also acts on the other species (Couret et al., 2014).

For FR2, individual larval weight and yield were positively affected by nutrient concentration at all four larval densities. Several authors observed that BSF larvae fed a diet high in protein and fat had a higher growth rate than fed low‐protein and ‐fat diets (Nguyen et al., 2013; Oonincx et al., 2015b; Tschirner & Simon, 2015). Grasshopper nymphs (*Schistocerca americana* Drury) fed diets with intermediate‐ to high‐nutrient contents were significantly larger than nymphs fed low‐nutrient diets (Hahn, 2005). However, high dietary fat content can also reduce BSF larval performance, either increasing the development time or reducing larval weight, ascribed to difficulties in processing fat (Spranghers et al., 2016). Considering highest larval weight as an indicator of dietary quality (Green et al., 2003), the NC3 diet was the most favourable diet composition tested.

In this study, higher densities positively affected individual larval weight at NC1 and NC2 in FR2. Larval aggregation produces higher temperatures that enhance food assimilation (Rivers & Dahlem, 2013). For instance, in the blowfly *P. regina*, larval aggregation has been reported to result in more efficient feeding, because the increased concentration of tryptic and alkaline excretions from groups of *P. regina* larvae may release more nutrients from the diet and allowed groups of larvae to forestall the toxic effects of some chemicals that interfere with conversion of amino acids and peptides into body mass, such as mimosine (Green et al., 2002). Probably, the alkaline excretions of groups of larvae neutralises the acidity associated with bacterial growth and pre‐digests the substrate (Green et al., 2003).

Overall, because our second experiment separated crowding per se from the effects of crowding via reduced food consumption, it appears that larval crowding positively affects larval performance. For instance, in view of the high survival rate observed for all densities and the enhanced overall larval performance at the highest densities, we conclude that overcrowding did not occur in BSF larvae. Parra Paz et al. (2015) found that larval density values of up to 5 larvae cm^−2^ do not significantly influence larval development time and weight as long as they are provided with an amount of food no lower than 1.7 g/larva (DM). It is possible that an overcrowding environment reduces final larval weight as reported for *Musca domestica* L. (Barnard & Geden, 1993).

BSF larvae on average have both a high‐protein and ‐fat content and their body composition depends on the quality of food ingested (Barragan‐Fonseca et al., 2017). In this study, we found that larval crude protein was affected by nutrient concentration, density, and their interaction in both feeding regimes. Larval crude protein content was similar in both feeding regimes and similar to values found in previous studies for larvae fed on chicken feed, by‐products, and manure (43.2 ± 2.7% DM) (Barragan‐Fonseca et al., 2017). Although there were significant differences in larval crude protein content among the three diets we tested, the absolute differences were small. Although larval body composition depended on diet quality, dietary protein content did not determine the protein content in the larval body. In our study, the low‐protein content (3.5%) in the NC1 diet resulted in a similarly high larval crude protein content as on the NC2 and NC3 diets. Indeed, FR2 resulted in the highest crude protein content in larvae at the lowest NC. This is in line with the findings of Tschirner & Simon (2015) who reported that crude protein was higher (52.3% DM) in larvae fed on the diet with the lowest crude protein content (8.5% DM). Spranghers et al. (2016) also found a similar protein content (ranging from 40 to 43% DM) in larvae fed on diets having protein contents ranging from 9 to 25% DM. In contrast, larvae fed on high‐protein substrates of animal origin, such as liver or meat, had much higher protein content (60.3 ± 3.3% DM) than larvae fed on vegetable waste (38.5% DM) (Nguyen et al., 2015).

Insect crude protein levels have commonly been calculated from total elemental nitrogen content using the standard nitrogen‐to‐protein conversion factor of 6.25. However, according to Janssen et al. (2017), this factor overestimates protein content, due to the presence of non‐protein nitrogen in insects. For instance, Diener et al. (2009) and Spranghers et al. (2016) reported that BSF larvae had between 5 and 20% lower crude protein content when corrected for chitin. Janssen et al. (2017) propose the conversion factor of 4.67 to be adopted for determining protein content of BSF larvae to avoid overestimation.

Larval crude fat content was also affected by nutrient concentration, density, and their interaction, and strong differences were observed between both feeding regimens. In FR1, overall crude fat content was lower than in FR2, likely due to food limitation. Larvae that did not reach the prepupal stage had the lowest crude fat contents and the lowest larval weights, which points to starvation of the larvae; thus, they had to mobilise their fat reserves (Tschirner & Simon, 2015), did not grow, and could not complete development. From a physiological perspective development time is an emergent property, because moulting and metamorphosis are triggered by size, not by time (Grunert et al., 2015). In FR2, the crude fat content was lower in larvae fed diet NC1, than in larvae fed on the NC2 and NC3 diets at all three densities. Larvae fed on NC2 and NC3 diets had similar fat content. These fat contents are in the range of values reported in the literature on BSF larvae and vary substantially among substrates (ranging from 7 to 39% DM) (Barragan‐Fonseca et al., 2017). For instance, substrates with high‐fat and carbohydrate contents increase larval crude fat content (Zheng et al., 2012b), whereas larvae fed on high‐fibre and/or low‐fat diets have lower crude fat content (3.4–6.6% DM) (Nguyen et al., 2015; Tschirner & Simon, 2015).

A striking finding of our work is the constancy of protein content, whereas fat content varies strongly. This means the contents of other body components must vary inversely with fat. The most likely candidate macronutrients for these are carbohydrates, present as mono‐ and disaccharides, glycogen, and chitin.

The body size at which a larva stops growing determines adult body size (Grunert et al., 2015). In BSF adult weight is positively correlated with adult fecundity – females with larger bodies had the largest ovaries and basal oocytes (Gobbi et al., 2013). In this study we also found that heavier BSF larvae had higher crude fat contents, which could be important for adult fitness (Andrewartha, 1952). Grasshopper nymphs (*S. americana*) fed diets with intermediate to high nutrient contents contained a significantly greater proportion of lipid stores in the adult stage, but not greater protein or carbohydrate reserves than individuals fed low‐nutrient content diets (Hahn, 2005).

In conclusion, our experiments demonstrated that larval density and nutrient concentration affect BSF performance and body composition. Moreover, larval protein content is regulated within narrow limits, whereas larval crude fat content is strongly affected by both nutrient concentration and larval density. Larger scale experiments are required to examine the extent to which results can be extrapolated to mass production conditions. Follow‐up experiments should also evaluate consequences for the adult stage as reproduction is a main factor in the continuity of an animal production system.

## Supporting information


**Table S1.** Statistical analysis with Generalised Linear Models (GLM; Wald χ^2^) and ANOVA (F) of various performance and body composition parameters of *Hermetia illucens* larvae under two feeding regimes (FR1 and 2), on three diets differing in nutrient concentration (NC), kept at four larval densities per container.

## References

[eea12716-bib-0001] Andrewartha H (1952) Diapause in relation to the ecology of insects. Biological Reviews 27: 50–107.

[eea12716-bib-0003] Barnard D & Geden C (1993) Influence of larval density and temperature in poultry manure on development of the house fly (Diptera: Muscidae). Environmental Entomology 22: 971–977.

[eea12716-bib-0004] Barragan‐Fonseca KB , Dicke M & van Loon JJA (2017) Nutritional value of the black soldier fly (*Hermetia illucens* L.) and its suitability as animal feed – a review. Journal of Insects as Food and Feed 3: 105–120.

[eea12716-bib-0005] Coelho AR , Dinis MT & Reis J (2013) Effect of diet and stocking densities on life history traits of *Clea helena* (Philippi 1847) reared in captivity. Journal of Aquaculture and Research Development 4: 187.

[eea12716-bib-0006] Couret J , Dotson E & Benedict MQ (2014) Temperature, larval diet, and density effects on development rate and survival of *Aedes aegypti* (Diptera: Culicidae). PLoS ONE 9: e87468. 24498328 10.1371/journal.pone.0087468 PMC3911954

[eea12716-bib-0007] Diener S , Zurbrügg C & Tockner K (2009) Conversion of organic material by black soldier fly larvae: establishing optimal feeding rates. Waste Management & Research 27: 603–610. 19502252 10.1177/0734242X09103838

[eea12716-bib-0008] Diener S , Studt Solano N , Roa Gutiérrez F , Zurbrügg C & Tockner K (2011a) Biological treatment of municipal organic waste using black soldier fly larvae. Waste and Biomass Valorization 2: 357–363.

[eea12716-bib-0009] Diener S , Zurbrügg C , Gutiérrez FR , Nguyen DH , Morel A et al. (2011b) Black soldier fly larvae for organic waste treatment – prospects and constraints Proceedings of the WasteSafe – 2nd International Conference on Solid Waste Management in the Developing Countries (ed. by Alamgir M), pp. 52–59. Khulna University of Engineering and Technology, Khulna, Bangladesh.

[eea12716-bib-0010] Fatchurochim S , Geden C & Axtell R (1989) Filth fly (Diptera) oviposition and larval development in poultry manure of various moisture levels. Journal of Entomological Science 24: 224–231.

[eea12716-bib-0011] Gobbi P , Martínez‐Sánchez A & Rojo S (2013) The effects of larval diet on adult life‐history traits of the black soldier fly, *Hermetia illucens* (Diptera: Stratiomyidae). European Journal of Entomology 110: 461–468.

[eea12716-bib-0012] Green TR & Popa R (2012) Enhanced ammonia content in compost leachate processed by black soldier fly larvae. Applied Biochemistry and Biotechnology 166: 1381–1387. 22238016 10.1007/s12010-011-9530-6

[eea12716-bib-0013] Green PW , Simmonds MS & Blaney WM (2002) Does the size of larval groups influence the effect of metabolic inhibitors on the development of *Phormia regina* (Diptera: Calliphoridae) larvae? European Journal of Entomology 99: 19–22.

[eea12716-bib-0014] Green PW , Simmonds MS & Blaney WM (2003) Diet nutriment and rearing density affect the growth of black blowfly larvae, *Phormia regina* (Diptera: Calliphoridae). European Journal of Entomology 100: 39–42.

[eea12716-bib-0015] Grunert LW , Clarke JW , Ahuja C , Eswaran H & Nijhout HF (2015) A quantitative analysis of growth and size regulation in *Manduca sexta*: the physiological basis of variation in size and age at metamorphosis. PLoS ONE 10: e0127988. 26011714 10.1371/journal.pone.0127988 PMC4444085

[eea12716-bib-0016] de Haas EM , Wagner C , Koelmans AA , Kraak MHS & Admiraal W (2006) Habitat selection by chironomid larvae: fast growth requires fast food. Journal of Animal Ecology 75: 148–155. 16903052 10.1111/j.1365-2656.2005.01030.x

[eea12716-bib-0017] Hahn DA (2005) Larval nutrition affects lipid storage and growth, but not protein or carbohydrate storage in newly eclosed adults of the grasshopper *Schistocerca americana* . Journal of Insect Physiology 51: 1210–1219. 16098985 10.1016/j.jinsphys.2005.06.011

[eea12716-bib-0018] Harnden LM & Tomberlin JK (2016) Effects of temperature and diet on black soldier fly, *Hermetia illucens* (L.) (Diptera: Stratiomyidae), development. Forensic Science International 266: 109–116. 27236368 10.1016/j.forsciint.2016.05.007

[eea12716-bib-0019] Holmes LA (2010) Role of Abiotic Factors on the Development and Life History of the Black Soldier Fly, Hermetia illucens (L.) (Diptera: Stratiomyidae). MSc Thesis, University of Windsor, Windsor, ON, Canada.

[eea12716-bib-0021] ISO 5983‐1 (2005) Animal Feeding Stuffs. Determination of Nitrogen Content and Calculation of Crude Protein Content. International Organization for Standardization, Geneva, Switzerland.

[eea12716-bib-0022] ISO 6492 (1999) Animal Feeding stuffs. Determination of Fat Content. International Organization for Standardization, Geneva, Switzerland.

[eea12716-bib-0023] Jannat KNE & Roitberg BD (2013) Effects of larval density and feeding rates on larval life history traits in *Anopheles gambiae* ss (Diptera: Culicidae). Journal of Vector Ecology 38: 120–126. 23701616 10.1111/j.1948-7134.2013.12017.x

[eea12716-bib-0024] Janssen RH , Vincken J‐P , van den Broek LA , Fogliano V & Lakemond CM (2017) Nitrogen‐to‐protein conversion factors for three edible insects: *Tenebrio molitor*,* Alphitobius diaperinus*, and *Hermetia illucens* . Journal of Agricultural and Food Chemistry 65: 2275–2278. 28252948 10.1021/acs.jafc.7b00471 PMC5364430

[eea12716-bib-0026] Lardé G (1989) Investigation on some factors affecting larval growth in a coffee‐pulp bed. Biological Wastes 30: 11–19.

[eea12716-bib-0028] Lihoreau M , Buhl J , Charleston MA , Sword GA , Raubenheimer D & Simpson SJ (2015) Nutritional ecology beyond the individual: a conceptual framework for integrating nutrition and social interactions. Ecology Letters 18: 273–286. 25586099 10.1111/ele.12406 PMC4342766

[eea12716-bib-0029] Liu Q , Tomberlin JK , Brady JA , Sanford MR & Yu Z (2008) Black soldier fly (Diptera: Stratiomyidae) larvae reduce *Escherichia coli* in dairy manure. Environmental Entomology 37: 1525–1530. 19161696 10.1603/0046-225x-37.6.1525

[eea12716-bib-0030] May B (1961) The occurrence in New Zealand and the life‐history of the soldier fly *Hermetia illucens* (L.) (Diptera: Stratiomyidae). New Zealand Journal of Science 4: 55–65.

[eea12716-bib-0031] Miller RS (1964) Larval competition in *Drosophila melanogaster* and *D. simulans* . Ecology 45: 132–148.

[eea12716-bib-0032] Moreau J , Benrey B & Thiéry D (2006) Assessing larval food quality for phytophagous insects: are the facts as simple as they appear? Functional Ecology 20: 592–600.

[eea12716-bib-0033] Myers HM , Tomberlin JK , Lambert BD & Kattes D (2008) Development of black soldier fly (Diptera: Stratiomyidae) larvae fed dairy manure. Environmental Entomology 59: 77–88. 10.1603/0046-225x(2008)37[11:dobsfd]2.0.co;2 18348791

[eea12716-bib-0034] Nguyen TT , Tomberlin JK & Vanlaerhoven S (2013) Influence of resources on *Hermetia illucens* (Diptera: Stratiomyidae) larval development. Journal of Medical Entomology 50: 898–906. 23926790 10.1603/me12260

[eea12716-bib-0035] Nguyen TT , Tomberlin JK & Vanlaerhoven S (2015) Ability of black soldier fly (Diptera: Stratiomyidae) larvae to recycle food waste. Environmental Entomology 44: 406–410. 26313195 10.1093/ee/nvv002

[eea12716-bib-0036] Nijhout H (2003) The control of body size in insects. Developmental Biology 261: 1–9. 12941617 10.1016/s0012-1606(03)00276-8

[eea12716-bib-0037] Oonincx DGAB , van Huis A & van Loon JJA (2015a) Nutrient utilisation by black soldier flies fed with chicken, pig, or cow manure. Journal of Insects as Food and Feed 1: 131–139.

[eea12716-bib-0038] Oonincx DGAB , van Broekhoven S , van Huis A & van Loon JJA (2015b) Feed conversion, survival and development, and composition of four insect species on diets composed of food by‐products. PLoS ONE 10: e0144601. 26699129 10.1371/journal.pone.0144601 PMC4689427

[eea12716-bib-0039] Parra Paz AS , Carrejo NS & Gómez Rodríguez CH (2015) Effects of larval density and feeding rates on the bioconversion of vegetable waste using black soldier fly larvae *Hermetia illucens* (L.), (Diptera: Stratiomyidae). Waste and Biomass Valorization 6: 1059–1065.

[eea12716-bib-0040] Rivers DB & Dahlem GA (2013) The Science of Forensic Entomology. Wiley‐Blackwell, Chichester, UK.

[eea12716-bib-0041] Scriber JM & Slansky F (1981) The nutritional ecology of immature insects. Annual Review of Entomology 26: 183–211.

[eea12716-bib-0042] Sheppard CD , Tomberlin JK , Joyce JA , Kiser BC & Sumner SM (2002) Rearing methods for the black soldier fly (Diptera: Stratiomyidae). Journal of Medical Entomology 39: 695–698. 12144307 10.1603/0022-2585-39.4.695

[eea12716-bib-0043] Simon P , Krüger R & Ribeiro P (2011) Influence of diets on the rearing of predatory flies of housefly larvae. Arquivo Brasileiro de Medicina Veterinária e Zootecnia 63: 1414–1420.

[eea12716-bib-0044] Slansky F & Scriber JM (1985) Food consumption and utilization Comprehensive Insect Physiology Biochemistry and Pharmacology, Vol. 4 (ed. by Kerkut GA & Gilbert LI), pp. 87–163. Pergamon Press, Oxford, UK.

[eea12716-bib-0045] Slansky F & Wheeler GS (1989) Compensatory increases in food consumption and utilization efficiencies by velvetbean caterpillars mitigate impact of diluted diets on growth. Entomologia Experimentalis et Applicata 51: 175–187.

[eea12716-bib-0046] Spranghers T , Ottoboni M , Klootwijk C , Ovyn A , Deboosere S et al. (2016) Nutritional composition of black soldier fly (*Hermetia illucens*) prepupae reared on different organic waste substrates. Journal of the Science of Food and Agriculture 97: 2594–2600. 27734508 10.1002/jsfa.8081

[eea12716-bib-0047] Sullivan RL & Sokal RR (1963) The effects of larval density on several strains of the house fly. Ecology 44: 120–130.

[eea12716-bib-0049] Tomberlin JK , Sheppard DC & Joyce JA (2002) Selected life‐history traits of black soldier flies (Diptera: Stratiomyidae) reared on three artificial diets. Annals of the Entomological Society of America 95: 379–386.

[eea12716-bib-0051] Tschirner M & Simon A (2015) Influence of different growing substrates and processing on the nutrient composition of black soldier fly larvae destined for animal feed. Journal of Insects as Food & Feed 1: 1–12.

[eea12716-bib-0052] Ujvari B , Wallman JF , Madsen T , Whelan M & Hulbert A (2009) Experimental studies of blowfly (*Calliphora stygia*) longevity: a little dietary fat is beneficial but too much is detrimental. Comparative Biochemistry and Physiology A 154: 383–388. 10.1016/j.cbpa.2009.07.012 19632351

[eea12716-bib-0053] Yoshioka M , Couret J , Kim F , McMillan J , Burkot TR et al. (2012) Diet and density dependent competition affect larval performance and oviposition site selection in the mosquito species *Aedes albopictus* (Diptera: Culicidae). Parasites & Vectors 5: 225. 23044004 10.1186/1756-3305-5-225 PMC3481443

[eea12716-bib-0054] Zheng L , Hou Y , Li W , Yang S , Li Q & Yu Z (2012a) Biodiesel production from rice straw and restaurant waste employing black soldier fly assisted by microbes. Energy 47: 225–229.

[eea12716-bib-0055] Zheng L , Qing L , Jibin Z & Ziniu Y (2012b) Double the biodiesel yield: rearing black soldier fly larvae, *Hermetia illucens*, on solid residual fraction of restaurant waste after grease extraction for biodiesel production. Renewable Energy 41: 75–79.

