# Supplementary material for: Influence of larval density and dietary nutrient concentration on performance, body protein, and fat contents of black soldier fly larvae (Hermetia illucens)
Source: Entomol Exp Appl. 2018 Sep 3;166(9):761–70. doi: 10.1111/eea.12716 (PMC6221057; doi:10.1111/eea.12716)
Supplement: Supplementary file 1 — Table S1. Statistical analysis with Generalised Linear Models (GLM; Wald χ2) and ANOVA (F) of various performance and body composition parameters of Hermetia illucens larvae under two feeding regimes (FR1 and 2), on three diets differing in nutrient concentration (NC), kept at four larval densities per container. [file EEA-166-761-s001.docx]

**Supporting Information**

**Table S1** Statistical analysis using Generalised Linear Models (GLM; Wald χ^2^) and ANOVA (F) of various performance and body composition parameters of *Hermetia illucens* larvae under two feeding regimes (FR1 and 2), on three diets differing in nutrient concentration (NC), kept at four larval densities per container

| Factors | | Density (D) | | |  | Nutrient concentration (NC) | | |  | D*NC | | |
| --- | --- | --- | --- | --- | --- | --- | --- | --- | --- | --- | --- | --- |
|  |  | Wald χ^2^ / F | d.f. | P |  | Wald χ^2^ / F | d.f. | P |  | Wald χ^2^ / F | d.f. | P |
| FR1 | Survival rate | 67.35 | 3 | <0.0001 |  | 5.221 | 2 | 0.064 |  | 37.21 | 6 | <0.0001 |
|  | Development time | 136.1 | 3 | <0.0001 |  | 773.5 | 2 | <0.0001 |  | 128.7 | 6 | <0.0001 |
|  | Individual larval weight | 909.01 | 3 | <0.0001 |  | 3127.5 | 2 | <0.0001 |  | 255.8 | 6 | <0.0001 |
|  | Larval yield | 3530.3 | 3 | <0.0001 |  | 9616.5 | 2 | <0.0001 |  | 2229.9 | 6 | <0.0001 |
|  | Larval crude protein | F = 6.33 | 3,24 | <0.0001 |  | F = 6.30 | 2,24 | <0.0001 |  | F = 2.7 | 6,24 | 0.039 |
|  | Larval crude fat | F = 30.2 | 3,24 | <0.0001 |  | F = 71.9 | 2,24 | <0.0001 |  | F = 2.9 | 6,24 | 0.026 |
| FR2 | Survival rate | F = 0.84 | 3,60 | 0.48 |  | F = 1.21 | 2,60 | 0.31 |  | F = 2.62 | 6,60 | 0.25 |
|  | Development time | 122.2 | 3 | <0.0001 |  | 108.1 | 2 | <0.0001 |  | 10.015 | 6 | 0.12 |
|  | Individual larval weight | 99.66 | 3 | <0.0001 |  | 3246.8 | 2 | <0.0001 |  | 2864 | 6 | <0.0001 |
|  | Larval yield | 8790.7 | 3 | <0.0001 |  | 1819.3 | 2 | <0.0001 |  | 795.6 | 6 | <0.0001 |
|  | Larval crude protein | F = 32.1 | 3,54 | <0.0001 |  | F = 8.5 | 2,54 | <0.0001 |  | F = 3.4 | 6,54 | 0.006 |
|  | Larval crude fat | F = 48.9 | 3,54 | <0.0001 |  | F = 67.1 | 2,54 | <0.0001 |  | F = 1.9 | 6,54 | 0.096 |
